# Common species link global ecosystems to climate change: dynamical evidence in the planktonic fossil record

**DOI:** 10.1098/rspb.2017.0722

**Published:** 2017-07-12

**Authors:** Bjarte Hannisdal, Kristian Agasøster Haaga, Trond Reitan, David Diego, Lee Hsiang Liow

**Affiliations:** 1 Centre for Geobiology, Department of Earth Science, University of Bergen, PO Box 7803, 5020 Bergen, Norway; 2 Bjerknes Centre for Climate Research, University of Bergen, PO Box 7803, 5020 Bergen, Norway; 3 Centre for Ecological and Evolutionary Synthesis, Department of Biosciences, University of Oslo, PO Box 1066, Blindern, 0316 Oslo, Norway; 4 Natural History Museum, University of Oslo, PO Box 1172 Blindern, 0318 Oslo, Norway

**Keywords:** global abundance, foraminifera, Cenozoic era, time series, causality

## Abstract

Common species shape the world around us, and changes in their commonness signify large-scale shifts in ecosystem structure and function. However, our understanding of long-term ecosystem response to environmental forcing in the deep past is centred on species richness, neglecting the disproportional impact of common species. Here, we use common and widespread species of planktonic foraminifera in deep-sea sediments to track changes in observed global occupancy (proportion of sampled sites at which a species is present and observed) through the turbulent climatic history of the last 65 Myr. Our approach is sensitive to relative changes in global abundance of the species set and robust to factors that bias richness estimators. Using three independent methods for detecting causality, we show that the observed global occupancy of planktonic foraminifera has been dynamically coupled to past oceanographic changes captured in deep-ocean temperature reconstructions. The causal inference does not imply a direct mechanism, but is consistent with an indirect, time-delayed causal linkage. Given the strong quantitative evidence that a dynamical coupling exists, we hypothesize that mixotrophy (symbiont hosting) may be an ecological factor linking the global abundance of planktonic foraminifera to long-term climate changes via the relative extent of oligotrophic oceans.

## 1. Introduction

True species richness can be elusive even in well-studied ecosystems, because most species are very rare, and relatively few species account for most of the total abundance [1,2]. For example, only approximately 1.4% of the estimated tree species account for half of the biomass of the Amazon forest and control the cycling of water, carbon and nutrients [3]. Similarly, a recent survey of eukaryotic plankton diversity in the global ocean found that approximately 0.24% of the taxa accounted for half of the total number of rDNA reads [4]. The relative richness of major trophic groups in the plankton is similar across ocean basins, and codiversification of protistan parasites, mutualistic symbionts and their hosts suggests an important role for ecological (‘biotic’) interactions in driving global plankton diversity [4,5]. Oceanographic (‘abiotic’) factors, on the other hand, such as differences in nutrient levels among ocean basins, are more clearly reflected in the abundance of major groups [4,5]. For example, the species richness of diatoms is comparable across nutrient-rich coastal waters and oligotrophic oceans, but their abundance is strikingly different in high- and low-nutrient settings [6]. These first-order patterns of richness and abundance in global eukaryotic plankton suggest that long-term environmental forcing of global abundance, such as an increase in the global abundance of low-nutrient adapted groups in response to an expansion of oligotrophic conditions, can be independent of any change in richness.

Major oceanographic regime changes, linked to climatic and tectonic processes, have occurred on geological time scales. Geological archives may thus provide insights into how global environmental changes have impacted ocean biomes, as preserved in the sedimentary record. Declining abundance of common species may have dramatic impacts on ecosystem functioning [7–9], with potential cascading effects onto biogeochemical cycling and biotic mitigation of environmental perturbations. However, efforts to link past climatic and oceanographic changes to planktonic biota in deep time have remained focused on temporal changes in species richness [10–12], and the importance of common species as tracers of past ecosystem response has been neglected.

We seek to redress the situation by targeting species that are globally common and widespread at any given time, using the Summed Common species Occurrence Rate (SCOR). SCOR sums the observed occupancy (the proportion of sampled sites at which a species is present and observed) across a species set and is driven by the most widespread species in the set [13]. In general, widespread species are also abundant [14], and a positive relationship between global abundance and occupancy is found in the global ocean plankton [4], including the planktonic foraminifera (electronic supplementary material, figure S1*a*). Here, we define commonness as high global site occupancy, and show that if the global abundance–occupancy relationship is positive (albeit noisy), then SCOR is a proxy for relative changes in global abundance integrated over the total set of species included. We do not consider local abundances or geographical ranges of individual species. A species contributes to SCOR only if and when it is widespread, which accommodates the geological transience of commonness; in the fossil record, species and higher taxa generally show a gradual expansion and contraction of their spatial extent of occurrence, being rare (restricted) in the early and late stages of their known stratigraphic range [15–17]. Furthermore, species that make up the bulk of the fossil record, and biostratigraphically useful species in particular, tend to be common and widespread.

Turning to the rich deep-sea sedimentary record of the Cenozoic era (0–65 Ma), we apply SCOR to global occurrences of planktonic foraminifera, a calcifying unicellular zooplankton group of central importance to our understanding of Cenozoic climate history. The taxonomy of planktonic foraminifera is based on test (shell) morphology, and despite the presence of cryptic diversity, the validity of extant morphological taxa is generally supported by ribosomal sequence data [18,19]. Global patterns of morphospecies richness of extant planktonic foraminifera have been linked to various environmental factors, including sea surface temperature, mixed layer depth, productivity, geography and ocean currents [20,21]. On Cenozoic time scales, fossil diversity dynamics in planktonic foraminifera have been linked to a combination of ecological traits and oxygen isotope records via statistical model selection [10], or correlated to changes in deep-sea sedimentation [22]. The dynamics of global commonness, on the other hand, have remained unexplored.

We propose that changes in the commonness of the most common species may represent a more tractable and meaningful measure of past ecosystem response to environmental forcing than those in the estimated total number of species. As a measure of potential physical forcing, we use a reconstruction of changes in deep-ocean temperature (DOT) throughout the Cenozoic [23]. We use the DOT record because it is a robust, globally meaningful signal of long-term changes, compatible with our integrated, global-scale analysis. Temperature proxy records for the surface ocean show considerable spatial heterogeneity [24] and would call for a spatially explicit analysis, which is not considered here. Geological proxy records are generally noisy mixtures of signals that represent multiple processes and are derived from a sedimentary record that is itself an active component of the Earth system. Any causal connection detected between proxy records would necessarily be indirect with respect to the underlying processes of interest. Nonetheless, the DOT proxy record of high-latitude and deep-water temperature reflects a set of interlinked, climate-related oceanographic variables, including changes in geographical and vertical thermal structure, considered to be important physical controls on the long-term evolution of planktonic foraminifera [10,22,25–27]. Here, we find evidence of a dynamical coupling between planktonic foraminifera SCOR and DOT using three conceptually very different methods for detecting causality in time series.

## 2. Material and methods

### (a) Data

Occurrences of macro- and microperforate planktonic foraminifera were retrieved from the Neptune Sandbox Berlin (NSB) database [28,29] (on 22 April 2015). SCOR was calculated using ⅓ Myr time bins, in order to maximize the number of temporal observations for time-series analysis, but our results also hold on a lower resolution of ½ Myr. In addition to a SCOR curve for 572 species in the original NSB taxonomy (updated in 2014 to match the taxonomic name list of the International Ocean Discovery Program), we calculated an ensemble of 20 SCOR curves that included from 81 to 100 of the most common species. To assess the robustness of SCOR to differences in taxonomic opinion, we established the set of 100 most common species using a different taxonomic scheme [30] and checked that the time intervals of commonness for these species in NSB were congruent with published stratigraphic ranges of each species after this taxonomic re-mapping [30–32] (electronic supplementary material, figure S2 and table S1).

DOT estimates were obtained from Cramer *et al*. [23], using the *T*_δ−SL_ record (based on subtracting New Jersey sea-level estimates from a benthic δ^18^O stack) for the interval 9–65 Ma, and the scaled δ^18^O record for the interval 0–9 Ma, with their interpolation at 0.1 Myr resolution. The SCOR and DOT data are available in the electronic supplementary material.

### (b) Summed common species occurrence rate

We treat the observation of a specific number of individuals as a Poisson-distributed variable with parameter *λ* in each time bin. The probability of finding an individual of species *i* in time bin *j* is then *p_ij_* = 1 − exp(−*λ_ij_*), and thus *λ_ij_* = −ln(1 − *p_ij_*). In practice, *p_ij_* is estimated as *y_ij_*/*n_j_*, where *y_ij_* is the number of sites in which species *i* is observed at time bin *j* and *n_j_* is the number of sites in that time bin where at least one of the species included in the analysis is observed. SCOR is the total density of a given set of *m_j_* species in a time bin,2.1
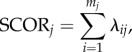
and we estimate its variance by the delta method [33],2.2
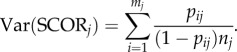


If species *i* occurs at all sites in time *j*, then *λ_ij_* is undefined. As *p* approaches 1, *λ* grows logarithmically, hence widespread species dominate SCOR.

### (c) Poseidon simulations

**Figure 1.**
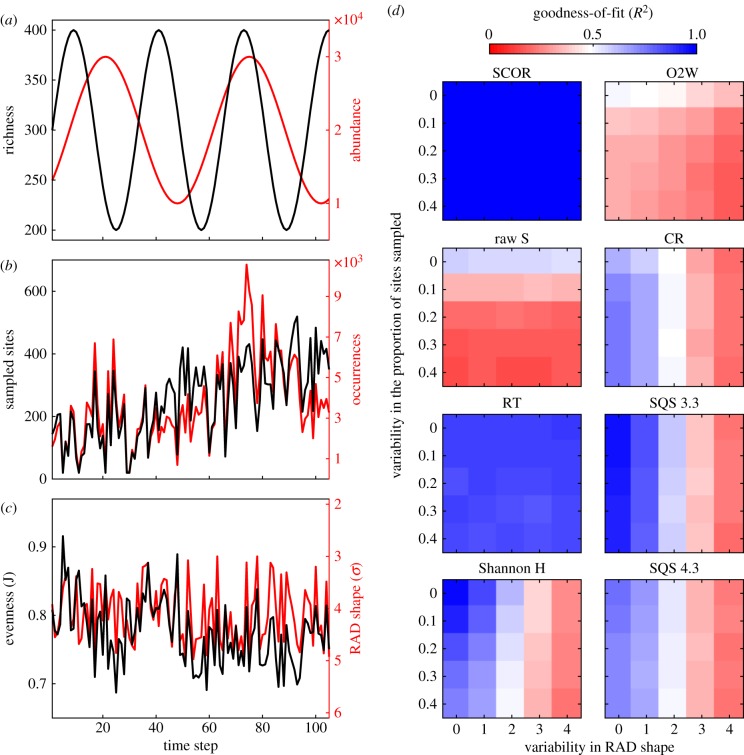
Performance of SCOR and richness estimators in Poseidon simulations. (*a*) Simulated species richness and total abundance are decoupled. (*b*) Sampled species occurrences are sensitive to the trend and short-term variability in sampled sites (in this example, variability = 0.1, corresponding to the standard deviation around the mean trend). (*c*) Sampled species evenness (Pielou's J) captures changes in the shape parameter *σ* of the RAD (in this example, variability = 2, corresponding to the range of *σ*), superimposed on richness fluctuations and a net decrease caused by the sampling trend. (*d*) Sensitivity to sampling variability and RAD shape variability. Values are median goodness of fit (*R*^2^) of 50 model runs, comparing SCOR to true abundance, and richness estimates to true richness. SQS 3.3 and Shannon H were calculated on sampled abundance, the other richness estimators and SCOR used only occurrences. See text for abbreviations.

Here, we evaluate the sensitivity of SCOR to changes in (i) spatial sampling completeness and (ii) the shape of the species rank-abundance distribution (RAD). Our simulation model (Poseidon) consists of a user-specified number of spatial cells (sites), number of time steps, total number of species (richness) and total number of individuals (abundance). In our simulations, we allow true richness and abundance to vary independently (figure 1*a*), to highlight the key point that SCOR is unaffected by changes in richness. In each time step, we distribute individuals randomly among species, obeying a lognormal RAD. The RAD shape parameter (*σ*) can be fixed or time-varying. We distribute individuals randomly among sites, and we sample only a proportion of the sites. This proportion increases from 0.1 to 0.4 through time (sampling coverage decreases with age), with any short-term sampling variability superimposed on this trend (figure 1*b*). Local preservation variability can be subsumed in the spatial sampling, because the quantities we focus on here are based on species occurrence (presence/absence) at sites, not on local abundance of individuals.

To test our proposition that commonness can be more tractable than richness, we compare the performance of SCOR to richness estimators commonly used in palaeobiology. Poseidon therefore calculates raw richness (S), range-through richness (RT; assuming a species existed in all time bins between its first and last occurrence), Shannon entropy (H), and several sampling-standardized richness metrics, including classic rarefaction (CR), occurrences-squared weighted (O2W) [34] and two versions of shareholder quorum subsampling [35,36]: SQS 3.3 (R script downloaded from the SQS website on 26 August 2014) and SQS 4.3 (perl script kindly provided by J. Alroy on 3 September 2014). We ran SQS 3.3 on sampled individuals (abundances) and SQS 4.3 on formatted Poseidon species occurrences, with a quorum level of 0.6 in all runs. Both SQS versions yielded very similar results when given the same type of data (abundances or occurrences). We used 100 iterations/trials in all subsampling methods (CR, O2W and SQS). Shannon H from the SQS 3.3 R script was used to calculate Pielou's J evenness. Goodness of fit between true and estimated richness, and between true abundance and SCOR, was measured by the coefficient of determination (*R*^2^) between time series. A link to the Poseidon R scripts is provided below.

### (d) Time-series analysis

If we take seriously the notion that climate-related oceanographic changes and planktonic ecosystems are interacting components of the Earth system, then we need to approach the possible long-term linkage between SCOR and DOT from a dynamical systems perspective. However, SCOR and DOT are both indirect proxy records that are not uniquely determined by any single physical or ecological process, and a dynamical coupling detectable in the proxy records would be a result of transitive chains of unobserved causal intermediates. In addition, the tectonic, climatic, atmospheric and biotic boundary conditions have all evolved over the Cenozoic. These challenges, combined with uncertainty in the relative importance and scales of mechanisms, limit the feasibility of explicit process modelling of a SCOR–DOT interaction throughout the Cenozoic. Instead, our approach is to test the hypothesis of a causal relationship using dynamical information inherent in the observed records, which enables us to advance beyond static correlations while avoiding unwarranted mechanistic assumptions.

We used three different time-series analysis methods to test for a causal relationship between SCOR and DOT: (i) convergent cross mapping (CCM) [37,38], based on the concept of state space reconstruction from time-delay embedding [39]; (ii) information transfer (IT) analysis [40,41], based on the concept of directional information flow [42]; and (iii) Bayesian inference of causal connections based on linear stochastic differential equations (SDEs) [43,44]. To distinguish unidirectional forcing from bidirectional causality, we calculated CCM for different time lags of the original time series. If there is a discernible delay between cause and effect, then CCM is expected to peak for negative time lags in the direction(s) of true causality (past predicts future). If true causality is unidirectional but with synchrony (inducing two-way predictability), then any CCM skill in the non-causal direction is expected to peak for positive lags (future predicts past). IT analysis has previously been applied to geological records [13,41,45]. Here, we expand on earlier applications by using time lags analogously to the lagged CCM. If there is a causal delay, then IT is expected to peak for negative time lags in the direction(s) of true causality (past → future), and any IT in the non-causal direction is expected to peak for positive lags. In the analysis of SCOR and DOT, lagged CCM and IT are both reported as median values and 95% ranges at each lag calculated by iterative sampling across an ensemble of SCOR curves, with significance evaluated against a distribution of surrogate time series. SDE analysis is a model-based approach. Here, we first determined the most probable stochastic models for SCOR and DOT separately, including possible hidden (unobserved) process layers, then we determined the most probable connections between these two models, distinguishing correlations from causal connections and inferring the relative strength, direction and response time of causal connections [43]. A more detailed description of each time-series analysis method is provided in the electronic supplementary material.

## 3. Results

### (a) Poseidon numerical experiments

We find that SCOR is robust to variability in both spatial sampling and RAD shape (figure 1*d*), as well as variability in the abundance–occupancy relationship (electronic supplementary material, figure S1). As expected, raw S decays rapidly with increasing sampling variability, but is insensitive to changes in RAD shape (*σ*). RT is relatively robust to both factors, as the species pool and/or sampling is sufficient to avoid severe edge effects. Shannon H reflects both richness and evenness, and, with increasing *σ* variability, H becomes more sensitive to *σ* than to changes in richness. O2W shows overall poor agreement with true richness. CR and SQS, being sampling standardization methods, are robust to the isolated effect of spatial sampling variability on richness, all else being equal. However, both CR and SQS are highly sensitive to changes in RAD shape. As with Shannon H, increasing *σ* variability causes CR and SQS to lose track of richness (figure 1*d*) and to respond to changes in *σ* instead (electronic supplementary material, figure S3). In Poseidon, *σ* variability is a white noise, entirely random with respect to both richness and abundance. Hence, the sensitivity of subsampling methods to RAD shape exists independently of how one might choose to measure sample evenness.

Estimates of SQS richness in the NSB data confirm the sensitivity to RAD shape found in Poseidon. Indeed, SQS richness can be reproduced by simply combining the raw richness and evenness curves, a relationship that holds for different taxa in NSB and across all Poseidon simulations (electronic supplementary material, figure S4). Changes in RAD shape are thus a significant confounding factor, at least for the specific subsampled richness algorithms tested here.

A data source like NSB, in which species occurrences were originally recorded for biostratigraphic purposes, is well suited for SCOR, because SCOR is only sensitive to common and widespread species, and biostratigraphically useful species are typically common and widespread. Nevertheless, we have included a Poseidon experiment where the recording of species (presence/absence) is random with respect to global commonness (electronic supplementary material, figure S5). Even in this scenario, which is designed to be particularly biased against it, SCOR still performs better than richness estimators. This experiment shows that we do not have to capture every abundant species for our approach to be meaningful.

### (b) Planktonic foraminifera summed common species occurrence rate

**Figure 2.**
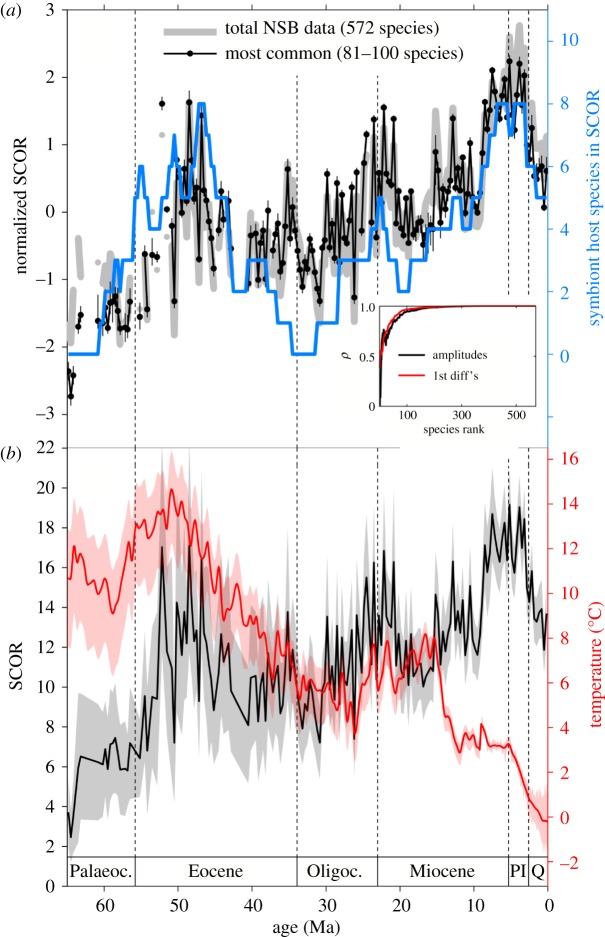
Cenozoic planktonic foraminifera SCOR and DOT records. (*a*) Normalized SCOR calculated at ⅓ Myr resolution, on total NSB data (grey) and on 20 subsets that included 81–100 of the most common species (ensemble mean in black, vertical bars represent the ensemble range). Blue line indicates the number of inferred symbiont-bearing species contributing to SCOR in each time bin (electronic supplementary material, figure S2). Inset: SCOR convergence on total curve with species added in decreasing rank order, measured as Pearson's *ρ* on amplitudes (black) and first differences (red). (*b*) Ensemble mean SCOR from (*a*) with 2 × s.e. confidence bounds (grey; electronic supplementary material) and reconstructed DOT at 0.1 Myr resolution with 95% confidence bounds [23]. Ma, million years before present; Palaeoc., Palaeocene; Oligoc., Oligocene; Pl, Pliocene; Q, Quaternary.

Planktonic foraminifera SCOR converges rapidly as species are added in rank order of decreasing commonness (figure 2*a*). The SCOR curve for the 572 original NSB species is statistically indistinguishable from the SCOR curve of the approximately 100 most common species, because SCOR is driven by the most common and widespread species. We based our final analyses on an ensemble of 20 SCOR curves, including from 81 to 100 of the most common species, after taxonomic remapping (electronic supplementary material, figure S2 and table S1). This SCOR ensemble was then compared with the Cenozoic DOT reconstruction (figure 2*b*) and subjected to a series of analyses designed to formally detect dynamical causal coupling.

### (c) Causal analyses

**Figure 3.**
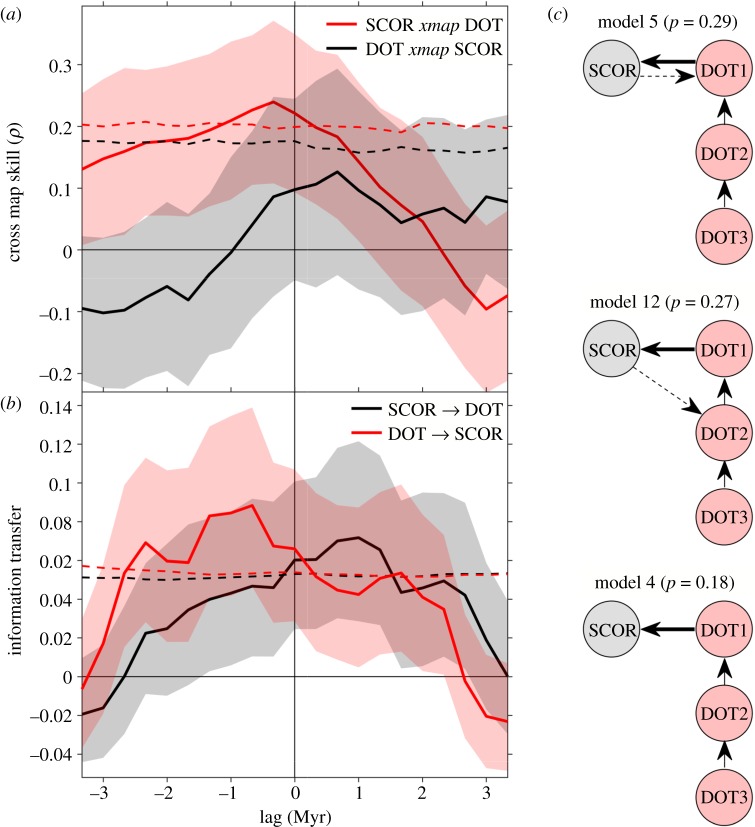
Causal directionality between SCOR and DOT over the Cenozoic. CCM skill (*a*) and IT (*b*) between SCOR and DOT as a function of time lag. If past DOT drives SCOR, then SCOR *xmap* DOT, and information flows DOT → SCOR, at negative lags. Values are means and 95% confidence bounds, dashed lines are 95th percentiles of surrogates (electronic supplementary material). All values are normalized to a surrogate mean of zero. (*c*) Schematic of the three most probable connection models in SDE analysis (electronic supplementary material, figure S6). Posterior probabilities of models are noted in parentheses. Circles represent process layers, and arrows indicate causal directionality. Stippled arrows indicate a much weaker influence per time unit (electronic supplementary material).

Although the net trends are inversely related, fluctuations in SCOR and DOT do not show consistent negative or positive covariation throughout the Cenozoic (figure 2*b*; for first-differenced data at zero lag, Spearman's *ρ* = 0.11, *p* = 0.13). However, CCM skill from foraminifera SCOR to DOT peaks at a negative lag, indicating that the SCOR signal carries a response to past changes captured in the DOT record (‘SCOR *xmap* DOT’; figure 3*a*). CCM skill in the opposite direction peaks at positive lags, but this skill is not significant relative to the surrogates (figure 3*a*). This result is consistent with a unidirectional forcing of SCOR by DOT, where predictability potentially goes both ways, but the causal direction peaks at negative lags [38]. IT analysis supports this causal directionality: predictive information flow is significant from past DOT to SCOR, peaking at a negative lag of two to four time bins (approx. 1 Myr; figure 3*b*). In the opposite direction, IT peaks at the corresponding positive lags, also consistent with unidirectional forcing. Both IT and CCM indicate a time-delayed coupling, but age model noise and the indirect nature of proxy records imply that the absolute magnitude of the lags cannot be given a strict physical interpretation. We therefore limit our interpretation to the sign of the peak lag to infer the directionality of the causal relationship.

Using a series of linear SDEs to model correlation and causality between the two records, we recover relatively strong evidence that SCOR responds to changes in DOT, with a time lag of 0.33–1.1 Myr, in agreement with the CCM and IT analyses. Linear SDE analyses on SCOR and DOT individually show that the best descriptions for SCOR and DOT are a single-layer and a three-layer model, respectively (electronic supplementary material). We allowed for the possibility of a two-way connection in a statistical sense, because both proxies derive from the deep-sea carbonate record, which is a responsive component of the climate system and could, in principle, impart a shared signal. Consequently, there are 15 possible connection models, including the null hypothesis of no relationship (electronic supplementary material, figure S6). Posterior model probabilities provide substantial evidence for a causal connection between SCOR and DOT (electronic supplementary material). In the two most probable models (figure 3*c*), although a two-way connection is statistically established, the influence of DOT on SCOR is overwhelmingly dominant (electronic supplementary material). The third most likely model is a simple one-way connection from DOT to SCOR (figure 3*c*), consistent with the overall causal inference.

## 4. Discussion

Occurrences in the NSB database stem from ocean drilling expedition shipboard data, and the presence of taxonomic and stratigraphic errors can be problematic for constructing range-based richness or phylogeny [30]. Our approach is much less sensitive to taxonomic and stratigraphic noise. Furthermore, we do not use local abundance data in NSB, which may be influenced by local fluxes and preservation, to reconstruct global abundance. Instead, SCOR is based only on global occupancy, integrated across widespread species, because globally widespread species are typically globally abundant (electronic supplementary material, figure S1*a*) [4]. As predicted from the hollow shape of species-abundance distributions, our results are reproducible with fewer than 100 of the most common species (figure 2*a*). The overwhelming majority of species in NSB thus do not contribute to SCOR, and the use of different taxonomic schemes does not affect our results. SCOR does not depend on knowing true times of speciation and extinction; a species only contributes to SCOR if/when it is widespread, and stratigraphic outliers or long tails of occurrences, erroneous or not, have no impact. Hence, we need not distinguish between a species having gone truly extinct and it having become a rare, ecological ‘ghost’, like the coccolithophore *Tergestiella*/*Cyclagelosphaera*, recently found living 54 Myr after its presumed extinction [46]. In addition, even if morphospecies do not conform strictly to biological species, they may represent successful phenotypes and/or traits that potentially link global abundance to ecosystem functioning. We use SCOR for its simplicity and consider it a starting point for more sophisticated occupancy-based approaches [47].

The planktonic foraminifera SCOR does not show positive covariation with a Cenozoic SCOR curve for the coccolithophores, another important calcifying plankton group. Instead, their raw patterns are negatively correlated (Spearman's *ρ* = −0.28) and first differences are not significantly correlated (*ρ* = 0.15, *p* > 0.06). Although preservation can affect the local relative abundance of these two groups in the sediment [48], it does not explain the different SCOR patterns, because expansion or contraction of the global extent of deep-sea carbonate deposition would cause the preserved global occupancy of both calcifying groups to increase or decrease in similar ways. Aspects of the Cenozoic coccolithophore SCOR pattern have been linked to proxy records of atmospheric CO_2_ [13] and suggest that these calcifying phytoplankton could thrive in a high-CO_2_ world [49]. Cooling and CO_2_ decline across the Eocene–Oligocene transition were accompanied by a lowering of the carbonate compensation depth, which has been attributed to changes in the supply of weathering products to the ocean [50]. Like the coccolithophores [13], however, planktonic foraminifera show declining SCOR across this transition, which is opposite to that expected if SCORs were biased upwards by enhanced deep-sea preservation. On the other hand, if species absence/presence were random with respect to global abundance, then SCOR would suffer from increased short-term volatility, but Poseidon experiments show that even under such conditions, SCOR can track relative changes in global abundance (electronic supplementary material, figure S5). For the 25 sites common to both databases, the age models for the NSB data may deviate from those in the DOT record, but the age models are not systematically offset (electronic supplementary material, figure S7); hence, the presence of age model noise should weaken any statistical associations and bias against our results. Finally, we note that although the number and global coverage of sites in the NSB data decrease with age, this temporal decay of spatial sampling coverage in itself does not create a systematic bias in SCOR (electronic supplementary material, figure S8).

The congruence of our three independent analyses provides strong quantitative evidence of a dynamical coupling between the observed global occupancy of planktonic foraminifera and past climatic and oceanographic changes captured in the DOT proxy record. A Cenozoic increase in ocean thermal stratification due to polar cooling has long been proposed as a secular abiotic forcing of plankton evolution [27], or even a ‘universal driver’ of planktonic cell size [26,51]. Assimilating a Cenozoic DOT proxy in models of planktonic foraminifera diversification has also been found to increase model support [52], on time scales comparable with the predictive lags found here. However, we caution against reducing the DOT–SCOR coupling to any single mechanism operating through the evolving biotic, tectonic and atmospheric boundary conditions of the Cenozoic. Rather than attributing process to temporal correlations or net trends, we use information inherent in the data to demonstrate a dynamical coupling, while avoiding mechanistic assumptions. The magnitude of the predictive lag cannot be interpreted as a direct, physical process–response delay, but accommodates an indirect, heterogeneous causal pathway between DOT and SCOR. Our results instead open up new questions on the long-term dynamics of commonness and its linkage to environmental change. As an example, we briefly consider the ecological characteristics of the species responsible for the SCOR pattern.

Planktonic foraminifera inferred to host symbionts in the open-ocean mixed layer (Ecogroup 1 in [30]) are among the most widespread species that contribute to high SCOR during intervals of relative DOT warmth (i.e. positive deviations from the net DOT trend), especially the early Eocene and the late Miocene–Pliocene (figure 2*a*; electronic supplementary material, figure S2). By contrast, deeper-dwelling species not known to host symbionts contribute most to SCOR during intervals of relative DOT cooling, such as the latest Eocene–Oligocene and, in the case of thermocline-dwelling species, the mid-Miocene. Symbiont-hosting (mixotrophic) rhizarian protists are surprisingly abundant in oligotrophic oceans today [53,54]. If oligotrophic realms expand with surface warming in the near future [55], then the global abundance of mixotrophs could increase. Greater global abundance of mixotrophs might enhance the efficiency of the biological carbon pump in the ocean, through net increases in body size and faster sinking rates [56]. Although the global biogeochemical impact of planktonic foraminifera may be limited, their outstanding fossil record makes them a prime candidate for studying a planktonic ecosystem response to global environmental changes in the past. Despite the opposite net trends, long-term fluctuations in SCOR and DOT show intervals of positive covariation, suggesting that planktonic foraminifera proliferated in periods of relative DOT warmth. To the extent that long-term patterns in the DOT record reflect Cenozoic mean states of surface water temperature (e.g. early Eocene warming followed by long-term Eocene cooling), and if, on average, warmer water conditions correspond to lower nutrient levels, then mixotrophy may have allowed the global abundance of planktonic foraminifera to increase in a warmer ocean. In the warm early Eocene, for example, oligotrophic open-ocean ecosystems may have reached mid- and high latitudes [57]. If higher global abundance of mixotrophic plankton was associated with greater efficiency of the biological carbon pump [56], then the SCOR pattern would be consistent with the sharpened δ^13^C depth gradients observed in the warm early–mid–Eocene [58], and the reduction of δ^13^C depth gradients from the warm Pliocene to the cooler Pleistocene [59]. We propose that mixotrophy has been an ecological factor linking the global abundance of planktonic foraminifera to the relative extent of oligotrophic oceans. Testing this hypothesis calls for spatially resolved proxy data and spatially explicit occupancy modelling, which are out of the scope of this study.

In this paper, we have: (i) highlighted the importance of widespread species as indicators of global ecosystem response; (ii) shown that a simple measure of global occupancy can be used to track changes in global abundance, because widespread species are typically globally abundant (i.e. commonness, as we define it); (iii) shown that in the fossil record, commonness is more tractable than richness, because the same fundamental ecological property that lends power to commonness (i.e. only a few species are abundant and widespread) can be a weak spot for richness estimators; (iv) used dynamical information in global-scale proxy records to rigorously test the coarse-grained hypothesis of a causal relationship between observed global occupancy of planktonic foraminifera and long-term climate changes captured in DOT records; and (v) based on the quantitative evidence for a causal connection, put forward a more fine-grained hypothesis on the possible role of mixotrophy that we hope deserves future scrutiny.

## Supplementary Material

Supplementary text, figures, and tables

## Supplementary Material

Supplementary data table

## Data accessibility

The datasets used in this article are included in the electronic supplementary material. The Poseidon code is available on GitHub (https://github.com/bhannis/Poseidon).

## References

[1] Preston FW 1948 The commonness, and rarity, of species. Ecology 29, 254–283. (10.2307/1930989)

[2] Connolly SR et al. 2014 Commonness and rarity in the marine biosphere. Proc. Natl Acad. Sci. USA 111, 8524–8529. (10.1073/pnas.1406664111) 24912168 PMC4060690

[3] Steege ter H et al. 2013 Hyperdominance in the Amazonian tree flora. Science 342, 1243092 (10.1126/science.1243092) 24136971

[4] de Vargas C et al. 2015 Eukaryotic plankton diversity in the sunlit ocean. Science 348, 121605 (10.1126/science.1261605) 25999516

[5] Lima-Mendez G et al. 2015 Determinants of community structure in the global plankton interactome. Science 348, 1262073 (10.1126/science.1262073) 25999517

[6] Malviya S et al. 2016 Insights into global diatom distribution and diversity in the world's ocean. Proc. Natl Acad. Sci. USA 113, E1516–E1525. (10.1073/pnas.1509523113) 26929361 PMC4801293

[7] Gaston KJ 2011 Common ecology. Bioscience 61, 354–362. (10.1525/bio.2011.61.5.4)

[8] Dirzo R, Young HS, Galetti M, Ceballos G, Isaac NJB, Ben C 2014 Defaunation in the Anthropocene. Science 345, 401–406. (10.1126/science.1251817) 25061202

[9] Winfree R, Fox JW, Williams NM, Reilly JR, Cariveau DP 2015 Abundance of common species, not species richness, drives delivery of a real-world ecosystem service. Ecol. Lett. 18, 626–635. (10.1111/ele.12424) 25959973

[10] Ezard THG, Aze T, Pearson PN, Purvis A 2011 Interplay between changing climate and species' ecology drives macroevolutionary dynamics. Science 332, 349–351. (10.1126/science.1203060) 21493859

[11] Lazarus D, Barron J, Renaudie J, Diver P, Türke A 2014 Cenozoic planktonic marine diatom diversity and correlation to climate change. PLoS ONE 9, e84857 (10.1371/journal.pone.0084857.s007) 24465441 PMC3898954

[12] Falkowski PG 2004 The evolution of modern eukaryotic phytoplankton. Science 305, 354–360. (10.1126/science.1095964) 15256663

[13] Hannisdal B, Henderiks J, Liow LH 2012 Long-term evolutionary and ecological responses of calcifying phytoplankton to changes in atmospheric CO_2_. Glob. Change Biol. 18, 3504–3516. (10.1111/gcb.12007)

[14] Gaston KJ, Blackburn TM, Greenwood JJD, Gregory RD, Quinn RM, Lawton JH 2000 Abundance–occupancy relationships. J. Appl. Ecol. 37, 39–59. (10.1046/j.1365-2664.2000.00485.x)

[15] Foote M 2007 Symmetric waxing and waning of marine invertebrate genera. Paleobiology 33, 517–529. (10.1666/06084.1)

[16] Liow LH, Stenseth NC 2007 The rise and fall of species: implications for macroevolutionary and macroecological studies. Proc. R. Soc. B 274, 2745–2752. (10.1098/rspb.2000.1219) PMC2279224 17711843

[17] Liow LH, Skaug HJ, Ergon T, Schweder T 2010 Global occurrence trajectories of microfossils: environmental volatility and the rise and fall of individual species. Paleobiology 36, 224–252. (10.1666/08080.1)

[18] Morard R et al. 2015 PFR 2: a curated database of planktonic foraminifera 18S ribosomal DNA as a resource for studies of plankton ecology, biogeography and evolution. Mol. Ecol. Resour. 15, 1472–1485. (10.1111/1755-0998.12410) 25828689

[19] André A, Quillévéré F, Morard R, Ujiié Y, Escarguel G, De Vargas C, de Garidel-Thoron T, Douady CJ 2014 SSU rDNA divergence in planktonic foraminifera: molecular taxonomy and biogeographic implications. PLoS ONE 9, e104641 (10.1371/journal.pone.0104641) 25119900 PMC4131912

[20] Rutherford S, D'Hondt S, Prell W 1999 Environmental controls on the geographic distribution of zooplankton diversity. Nature 400, 749–753. (10.1038/23449)

[21] Fenton IS, Pearson PN, Dunkley Jones T, Farnsworth A, Lunt DJ, Markwick P, Purvis A 2016 The impact of Cenozoic cooling on assemblage diversity in planktonic foraminifera. Phil. Trans. R. Soc. B. 371, 20150224 (10.1098/rstb.2015.0224) 26977064 PMC4810817

[22] Peters SE, Kelly DC, Fraass AJ 2013 Oceanographic controls on the diversity and extinction of planktonic foraminifera. Nature 493, 398–401. (10.1038/nature11815) 23302802

[23] Cramer BS, Miller KG, Barrett PJ, Wright JD 2011 Late Cretaceous–Neogene trends in deep ocean temperature and continental ice volume: reconciling records of benthic foraminiferal geochemistry (δ^18^O and Mg/Ca) with sea level history. J. Geophys. Res. 116, C12023 (10.1029/2011JC007255)

[24] Veizer J, Prokoph A 2015 Temperatures and oxygen isotopic composition of Phanerozoic oceans. Earth-Sci. Rev. 146, 92–104. (10.1016/j.earscirev.2015.03.008)

[25] Cifelli R 1969 Radiation of Cenozoic planktonic foraminifera. Syst. Biol. 18, 154–168. (10.2307/2412601)

[26] Schmidt DN, Thierstein HR, Bollmann J, Schiebel R 2004 Abiotic forcing of plankton evolution in the Cenozoic. Science 303, 207–210. (10.1126/science.1090592) 14716007

[27] Lipps JH 1970 Plankton evolution. Evolution 24, 1 (10.2307/2406711) 28563010

[28] Lazarus D 1994 Neptune: a marine micropaleontology database. Math. Geol. 26, 817–832. (10.1007/BF02083119)

[29] Spencer-Cervato C 1999 The Cenozoic deep sea microfossil record: explorations of the DSDP/ODP sample set using the Neptune database. Palaeontol. Electron. 42, 1–268. (10.1080/00206810009465112)

[30] Aze T, Ezard THG, Purvis A, Coxall HK, Stewart DRM, Wade BS, Pearson PN 2011 A phylogeny of Cenozoic macroperforate planktonic foraminifera from fossil data. Biol. Rev. 86, 900–927. (10.1111/j.1469-185X.2011.00178.x) 21492379

[31] Stewart D, Pearson PN 2000 PLANKRANGE: a database of planktonic foraminiferal ranges. See http://palaeo.gly.bris.ac.uk/Data/plankrange.html.

[32] Pearson PN, Olsson RK, Huber BT, Hemleben C, Berggren WA (eds). 2006 Atlas of Eocene planktonic foraminifera. Fredericksburg, VA: Cushman Foundation for Foraminiferal Research.

[33] Casella G, Berger RL 2002 Statistical inference, 2nd edn Belmont, CA: Duxbury Press.

[34] Alroy J 2000 New methods for quantifying macroevolutionary patterns and processes. Paleobiology 26, 707–733. (10.1666/0094-8373(2000)026%3C0707:NMFQMP%3E2.0.CO;2)

[35] Alroy J 2010 The shifting balance of diversity among major marine animal groups. Science 329, 1191–1194. (10.1126/science.1189910) 20813951

[36] Alroy J 2014 Accurate and precise estimates of origination and extinction rates. Paleobiology 40, 374–397. (10.1666/13036)

[37] Sugihara G, May R, Ye H, Hsieh CH, Deyle E, Fogarty M, Munch S 2012 Detecting causality in complex ecosystems. Science 338, 496–500. (10.1126/science.1227079) 22997134

[38] Ye H, Deyle ER, Gilarranz LJ, Sugihara G 2015 Distinguishing time-delayed causal interactions using convergent cross mapping. Sci. Rep. 5, 1–9. (10.1038/srep14750) PMC4592974 26435402

[39] Takens F 1981 Detecting strange attractors in turbulence. In Dynamical systems and turbulence (eds Rand DA, Young LS), pp. 366–381. Berlin, Germany: Springer.

[40] Verdes P 2005 Assessing causality from multivariate time series. Phys. Rev. E 72, 026222 (10.1103/PhysRevE.72.026222) 16196699

[41] Hannisdal B 2011 Non-parametric inference of causal interactions from geological records. Am. J. Sci. 311, 315–334. (10.2475/04.2011.02)

[42] Schreiber T 2000 Measuring information transfer. Phys. Rev. Lett. 85, 461–464. (10.1103/PhysRevLett.85.461) 10991308

[43] Reitan T, Schweder T, Henderiks J 2012 Phenotypic evolution studied by layered stochastic differential equations. Ann. Appl. Stat. 6, 1531–1551. (10.1214/12-AOAS559)

[44] Liow LH, Reitan T, Harnik PG 2015 Ecological interactions on macroevolutionary time scales: clams and brachiopods are more than ships that pass in the night. Ecol. Lett. 18, 1030–1039. (10.1111/ele.12485) 26293753

[45] Hannisdal B, Peters SE 2011 Phanerozoic Earth system evolution and marine biodiversity. Science 334, 1122–1124. (10.1126/science.1210695) 22116884

[46] Hagino K, Young JR, Bown PR, Godrijan J, Kulhanek DK, Kogame K, Horiguchi T 2015 Re-discovery of a ‘living fossil’ coccolithophore from the coastal waters of Japan and Croatia. Mar. Micropaleontol. 116, 28–37. (10.1016/j.marmicro.2015.01.002)

[47] MacKenzie DI, Nichols JD, Royle JA, Pollock KH 2006 Occupancy estimation and modeling: inferring patterns and dynamics of species occurrence. New York, NY: Academic Press.

[48] Broecker W, Clark E 2009 Ratio of coccolith CaCO_3_ to foraminifera CaCO_3_ in late Holocene deep sea sediments. Paleoceanography 24, 260 (10.1029/2009PA001731)

[49] Rivero-Calle S, Gnanadesikan A, Del Castillo CE, Balch WM, Guikema SD 2015 Multidecadal increase in North Atlantic coccolithophores and the potential role of rising CO_2_. Science 350, 1533–1537. (10.1126/science.aaa8026) 26612836

[50] Pälike H, Lyle MW, Nishi H, Raffi I, Ridgwell A, Gamage K, Klaus A, Acton G, Anderson L 2012 A Cenozoic record of the equatorial Pacific carbonate compensation depth. Nature 488, 609–614. (10.1038/nature11360) 22932385

[51] Finkel ZV, Sebbo J, Feist-Burkhardt S 2007 A universal driver of macroevolutionary change in the size of marine phytoplankton over the Cenozoic. Proc. Natl Acad. Sci. USA 104, 20 416–20 420. (10.1073/pnas.0709381104) PMC2154445 18077334

[52] Ezard THG, Purvis A 2016 Environmental changes define ecological limits to species richness and reveal the mode of macroevolutionary competition. Ecol. Lett. 19, 899–906. (10.1111/ele.12626) 27278857 PMC4999050

[53] Biard T et al. 2016 In situ imaging reveals the biomass of giant protists in the global ocean. Nature 532, 504–507. (10.1038/nature17652) 27096373

[54] Guidi L et al. 2016 Plankton networks driving carbon export in the oligotrophic ocean. Nature 532, 465–470. (10.1038/nature16942) 26863193 PMC4851848

[55] Polovina JJ, Dunne JP, Woodworth PA, Howell EA 2011 Projected expansion of the subtropical biome and contraction of the temperate and equatorial upwelling biomes in the North Pacific under global warming. ICES J. Mar. Sci. 68, 986–995. (10.1093/icesjms/fsq198)

[56] Ward BA, Follows MJ 2016 Marine mixotrophy increases trophic transfer efficiency, mean organism size, and vertical carbon flux. Proc. Natl Acad. Sci. USA 113, 2958–2963. (10.1073/pnas.1517118113) 26831076 PMC4801304

[57] Lyle M, Barron J, Bralower TJ, Huber M, Olivarez Lyle A, Ravelo AC, Rea DK, Wilson PA 2008 Pacific Ocean and Cenozoic evolution of climate. Rev. Geophys. 46, RG2002 (10.1029/2005RG000190)

[58] John EH, Pearson PN, Coxall HK, Birch H, Wade BS, Foster GL 2013 Warm ocean processes and carbon cycling in the Eocene. Phil. Trans. R. Soc. A 371, 20130099 (10.1098/rsta.2013.0099) 24043871

[59] Schueth JD, Bralower TJ 2015 The relationship between environmental change and the extinction of the nannoplankton *Discoaster* in the early Pleistocene. Paleoceanography 30, 863–876. (10.1002/2015PA002803)

