# Supplementary material for: Common species link global ecosystems to climate change: dynamical evidence in the planktonic fossil record
Source: Proc Biol Sci. 2017 Jul 12;284(1858):20170722. doi: 10.1098/rspb.2017.0722 (PMC5524498; doi:10.1098/rspb.2017.0722)
Supplement: Supplementary text, figures, and tables [file rspb20170722supp1.pdf]

Electronic supplementary material

Common species link global ecosystems to climate change: dynamical evidence in the planktonic fossil record

Bjarte Hannisdal, Kristian Agasøster Haaga, Trond Reitan, David Diego, and Lee Hsiang Liow

Proceedings of the Royal Society B 20170722
http://dx.doi.org/10.1098/rspb.2017.0722

1. Supplementary Text

Time series analysis

We used two non-parametric and one model-based time series analysis method to test for causal directionality. For non-parametric time-lag analyses, missing SCOR values were linearly interpolated, and the DOT data averaged in the SCOR time bins. Furthermore, to assess significance with surrogate data, both records were detrended using a second-order polynomial to satisfy a stationarity criterion (Kwiatkowski et al., 1992), then normalized to zero mean and unit standard deviation. For consistency, the model-based analysis was also performed on the pre-processed data. However, the detrending may remove components of the variation relevant to uncovering the parameters of underlying processes. We therefore repeated the model-based analysis on untransformed data.

Convergent cross mapping (CCM)

If a time series X is a component of a dynamical system, we can construct a delay-coordinate embedding of the state space of the system into an m-dimensional real space, by constructing the vectors

$$E_X = \{\langle(t_i), x(t_i - \tau), x(t_i - 2\tau), \ldots, x(t_i - (m\tau)\rangle\}, \quad [1]$$

where $x(t_i)$ is the scalar value of the time series X at time t_i (Takens, 1981). That is, the vectors in E_X are in one-to-one correspondence with the states of the whole system. If X and Y are coupled variables of the same dynamical system (i.e. they are causally connected), this correspondence is also true for time series Y, and therefore E_X and E_Y are in one-to-one correspondence with each other. CCM uses this result to predict scalar values of Y from the coordinate-delay embedding of X and vice versa.

The CCM algorithm (Sugihara et al., 2012) locates, for each scalar point P_i in the prediction set (subset of time series Y), the contemporaneous state vector L_i in the library set (subset of state vectors in E_X). Next, it finds L_i's closest neighbors and estimates a value for the predictee P_i^* using simplex projection (Sugihara and May, 1990). CCM skill is determined by the correlation (Pearson's ρ) between estimated P_i^* and actual values of P_i. With increasing library size, CCM skill is expected to increase and converge if the variables are causally related. The notation "X *xmap* Y" refers to estimating $y(t_i)$ from corresponding lagged-coordinate state versions of $x(t_i)$, which is interpreted as "Y is causally influencing X".

We performed CCM using the rEDM package (Ye et al., 2015a), with embedding dimension $m = 2$, delay time step $\tau = 1$, and using the entire time series as both prediction and library sets. We used leave-one-out cross validation (i.e. the predictee P_i itself and points in a time radius of E around P_i were excluded from the libraries, such that no points sharing coordinates with P_i were used in the predictions Ye et al., 2015a). To distinguish unidirectional forcing from bidirectional causality, we calculated CCM for different time lags of the original time series. If there is a discernable delay between cause and effect, then CCM is expected to peak for negative time lags in the direction(s) of true causality (past predicts future). If true causality is unidirectional but with synchrony (inducing two-way predictability), then any CCM skill in the non-causal direction is expected to peak for positive lags (Ye et al., 2015b). Lagged CCM analysis of SCOR and DOT is reported as median CCM skill and 95% ranges at each lag for 300 iterated samples of library size 150. CCM is considered significant if it exceeds the 95th percentile of 300 amplitude-adjusted Fourier transform (AAFT) surrogate time series (Theiler et al., 1992).

Information transfer (IT)

If two processes X and Y are independent, then knowing the past l states of Y has no influence on the state transition probability of X, from x_n to x_{n+1}, beyond knowing the past k states of X alone:

$$p(x_{n+1}|x_n^{(k)}, y_n^{(l)}) = p(x_{n+1}|x_n^{(k)}). \quad [2]$$

Transfer entropy (Schreiber, 2000) quantifies the information lost when assuming independence by means of a Kullback-Leibler divergence:

$$T_{Y\to X} = \sum_{x,y} p(x_{n+1}, x_n^{(k)}, y_n^{(l)}) \log \frac{p(x_{n+1}|x_n^{(k)}, y_n^{(l)})}{p(x_{n+1}|x_n^{(k)})}, \quad [3]$$

which yields a non-symmetric measure of information flow, equivalent to Granger causality for linear, Gaussian systems (Barnett et al., 2009).

We implement transfer entropy in the modified IT form (Verdes, 2005; Hannisdal, 2011) and introduce time lags analogously to the lagged CCM. If there is a causal delay, then IT is expected to peak for negative time lags in the direction(s) of true causality (past $\to$ future). If true causality is unidirectional but with synchrony, then any IT in the non-causal direction is expected to peak for positive lags. IT varies as a function of the coarse-graining resolution, integrated in a single IT value using the area under the resulting curve (Verdes, 2005). Lagged IT analysis of SCOR and DOT is reported as median IT and 95% ranges for 100 draws of 100 random samples at each lag. IT is considered significant if it exceeds the 95th percentile of 5,000 AAFT surrogates.

Stochastic Differential Equations (SDE)

Linear SDEs can be used to distinguish between correlation and Granger causality, including uni- and bidirectional causation, as well as hidden (unmeasured) processes that expand the space of possible connections (Reitan et al., 2012; Liow et al., 2015). A basic SDE is

$$dX = -\alpha_X(X - \mu_X)dt + \sigma_X dB_t^{(X)}, \quad [4]$$

which describes a mean-reverting Ornstein-Uhlenbeck process (OUP) X with systematic (dt) and stochastic (dB) terms, expectation μ_X, standard deviation $s_X = \sigma_X/\sqrt{2\alpha_X}$, and half-life $t_{1/2,X} = \log(2)/\alpha_X$. If $\alpha_X = 0$, then dX describes a Wiener process (WP, or random walk). A process Y_1 may have a hidden process (or layer) Y_2 folded into its systematic part, such that Y_1 fluctuates with a lagged response to the OUP Y_2, and if there is a causal connection, e.g. from Y_2 to X, we can write

$$\begin{aligned} dX &= -\alpha_X(X - \mu_X - \beta_{Y_2\to X}(Y_2 - \mu_X))dt + \sigma_X dB_t^{(X)} \\ dY_1 &= -\alpha_{Y_1}(Y_1 - Y_2)dt + \sigma_{Y_1} dB_t^{(Y_1)} \\ dY_2 &= -\alpha_{Y_2}(Y_2 - \mu_Y)dt + \sigma_{Y_2} dB_t^{(Y_2)} \end{aligned} \quad [5]$$

where $\beta_{Y_2\to X}$ describes the connection strength.

We first modeled SCOR and DOT separately with up to three layers (two hidden), each layer being WP or OUP. We excluded one-layered WP, which does not accept incoming causal links. We also excluded between-layer feedback loops, which were numerically intractable. All model parameters were assigned normal priors, with 95% prior ranges of $\mu_i \in (-1.96, 1.96)$, $\sigma_i \in (0.01, 1.0)$, $t_{1/2,i} \in (0.1, 50)$Myr, where i denotes the layer, $\beta \in (-2, 2)$ for causal connections, and $\rho \in (-0.96, 0.96)$ for correlations. We used MCMC-based importance sampling to estimate Bayesian model probabilities (Reitan et al., 2012).

SDE analysis of SCOR and DOT used 1/2 Myr time bins, with SCOR calculated on the total original NSB species list (done prior to taxonomic re-mapping). However, CCM and IT results did not change with bin size or species re-mapping, and SDE conclusions should also be robust. The best SCOR model was a one-layer OUP, while the best DOT model was three-layered with a lowermost WP, yielding 15 connection models, including the null model of no relationship (figure S6). We allowed for two-way causality because SCOR and DOT both derive from deep-sea carbonate sediments. We assigned 50% prior probability to the null, and distributed 50% evenly among the 14 connection models.

The posterior probability of the null ($P = 0.11$) yields a Bayes factor favoring a connection of $B = 7.9$, which is substantial evidence (Jeffreys, 1961). In the two best models (figure S6) the upper DOT process

(DOT1) affects SCOR positively while SCOR affects DOT1 or DOT2 negatively, but DOT processes react very slowly to changes in SCOR (table S2). Because SCOR changes more rapidly, the DOT response will be smoothed out, hence DOT affects SCOR much more strongly per time unit than vice versa. In the third best model ($P = 0.18$; figure S6) DOT1 affects SCOR unidirectionally, hence the evidence for feedback is weak ($B = 2.6$). SDE analysis thus supports at least one connection ($B = 7.9$); causality rather than correlation, given a connection ($B = 12.5$); and DOT influencing SCOR, given a connection ($B = 15.4$). Although the DOT-SCOR causality is indirect, SDE shows a direct connection, because data on causal intermediates are lacking.

SDE analysis on untransformed data (uDOT and uSCOR, both having three-layer models with a lowermost WP) yielded very strong evidence ($B = 73$) for a connection, supporting feedback between the top layers uDOT1 and uSCOR1 (table S2). However, we found strong support for cyclical behaviour (1.5 Myr period), consistent with internal, between-layer feedback in uDOT.

References

Barnett, L., A. B. Barrett, and A. K. Seth (2009). Granger causality and transfer entropy are equivalent for Gaussian variables. *Physical Review Letters 103*(23), 238701–4.

Hannisdal, B. (2011). Non-parametric inference of causal interactions from geological records. *American Journal of Science 311*(4), 315–334.

Jeffreys, H. (1961). *Theory of probability* (3 ed.). Oxford: Oxford University Press.

Kwiatkowski, D., P. C. B. Phillips, P. Schmidt, and Y. Shin (1992). Testing the null hypothesis of stationarity against the alternative of a unit root. *Journal of econometrics 54*(1-3), 159–178.

Liow, L. H., T. Reitan, and P. G. Harnik (2015). Ecological interactions on macroevolutionary time scales: clams and brachiopods are more than ships that pass in the night. *Ecology Letters 18*(10), 1030–1039.

Reitan, T., T. Schweder, and J. Henderiks (2012). Phenotypic evolution studied by layered stochastic differential equations. *The Annals of Applied Statistics 6*(4), 1531–1551.

Schreiber, T. (2000). Measuring information transfer. *Physical Review Letters 85*(2), 461–464.

Sugihara, G., R. May, H. Ye, C. h. Hsieh, E. Deyle, M. Fogarty, and S. Munch (2012). Detecting causality in complex ecosystems. *Science 338*(6106), 496–500.

Sugihara, G. and R. M. May (1990). Nonlinear forecasting as a way of distinguishing chaos from measurement error in time series. *Nature 344*(6268), 734–741.

Takens, F. (1981). Detecting strange attractors in turbulence. In D. A. Rand and L. S. Young (Eds.), *Dynamical Systems and Turbulence*, pp. 366–381. Berlin Heidelberg: Springer.

Theiler, J., S. Eubank, A. Longtin, and B. Galdrikian (1992). Testing for nonlinearity in time series: the method of surrogate data. *Physica D: Nonlinear Phenomena 58*(1-4), 77–94.

Verdes, P. (2005). Assessing causality from multivariate time series. *Physical Review E 72*(2), 026222.

Ye, H., G. Sugihara, E. R. Deyle, R. M. May, K. Swanson, and A. A. Tsonis (2015a). Reply to Luo et al.: Robustness of causal effects of galactic cosmic rays on interannual variation in global temperature. *Proceedings of the National Academy of Sciences of the United States of America 112*(34), E4640–E4641.

Ye, H., E. R. Deyle, L. J. Gilarranz, and G. Sugihara (2015b). Distinguishing time-delayed causal interactions using convergent cross mapping. *Scientific Reports 5*, 1–9.

2. Supplementary Figures

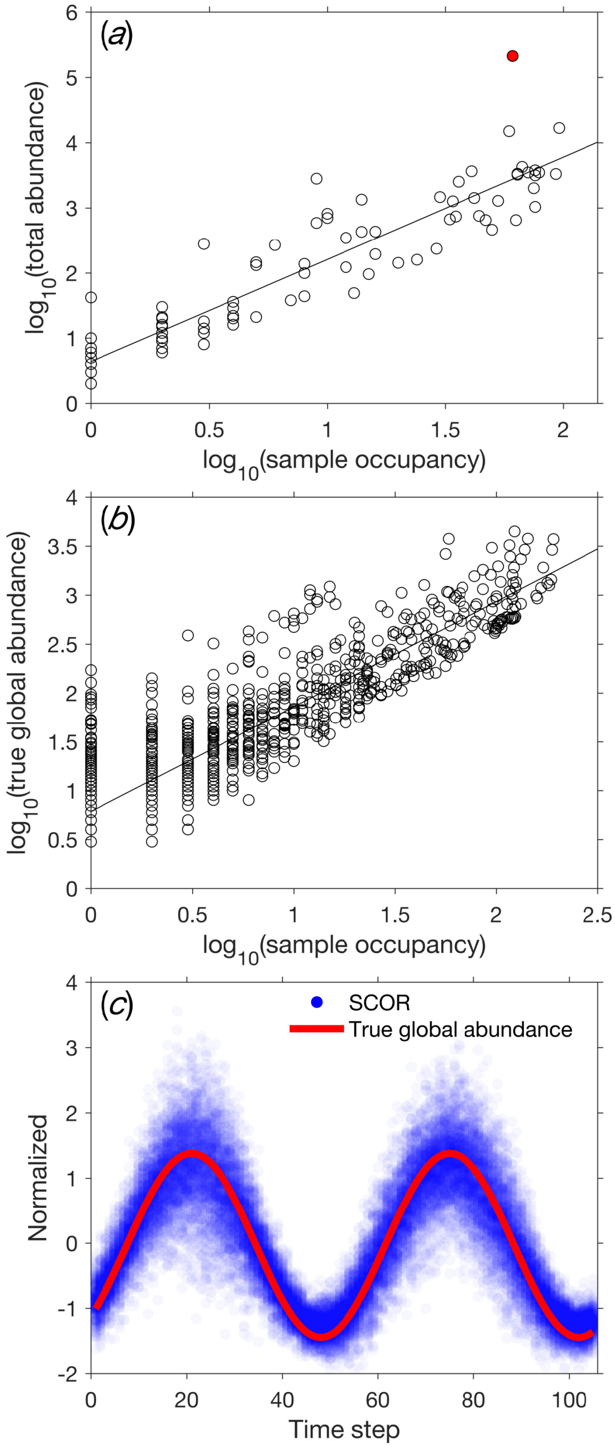

Figure S1. The relationship between global abundance and global occupancy is noisy, but positive. (*a*) Global abundance-occupancy relationship for modern planktonic foraminifera in the TARA Oceans data set (ref. 4). The data are metabarcodes (V9-18S rDNA sequences), their presence/absence across 334 sample localities, and the total abundance (number of reads) of each barcode. Barcodes were collapsed into "species" (OTUs, or "cid" identifiers). Here we include only those 102 species that have a planktonic taxon attribution showing >90% identity with a reference sequence, thus excluding 66 benthic, 8 tychopelagic, and 72 unresolved species (R. Morard, personal communication, Dec 19, 2016). The summed total abundance for each species is plotted against the number of sample localities in which it was present (sample occupancy). The 20 most widespread species account for more than 94% of the global abundance (or more than 84% if we exclude the most abundant species, marked in red). (*b*) Variability in the abundance-occupancy relationship in a Poseidon simulation. Parameter settings correspond to one of the scenarios in the random-loss experiment (figure S5), with RAD variability = 4 and a constant proportion of 50% of species randomly removed in each time step. This scenario matches the upper right-hand corner of the panels in figure S5*d*. The abundance-occupancy relationship is from a single time step (in which the spatial site sampling proportion is ∼16%), across ten model iterations. Like in the TARA Oceans data (*a*; see also ref. 4), there is greater variability above the trend line than below the trend line (abundant species are not always widespread, but widespread species are unlikely to be rare). (*c*) Random removal of species in each time step causes short-term volatility in SCOR, which is most severe in periods of high total abundance (depending on richness and RAD). Despite this volatility, SCOR (blue, encompassing 300 model iterations) is able to capture the relative changes in global abundance (red) with meaningful accuracy (time series are normalized to mean zero and unit standard deviation), and outperforms richness estimators under these conditions (figure S5*d*).

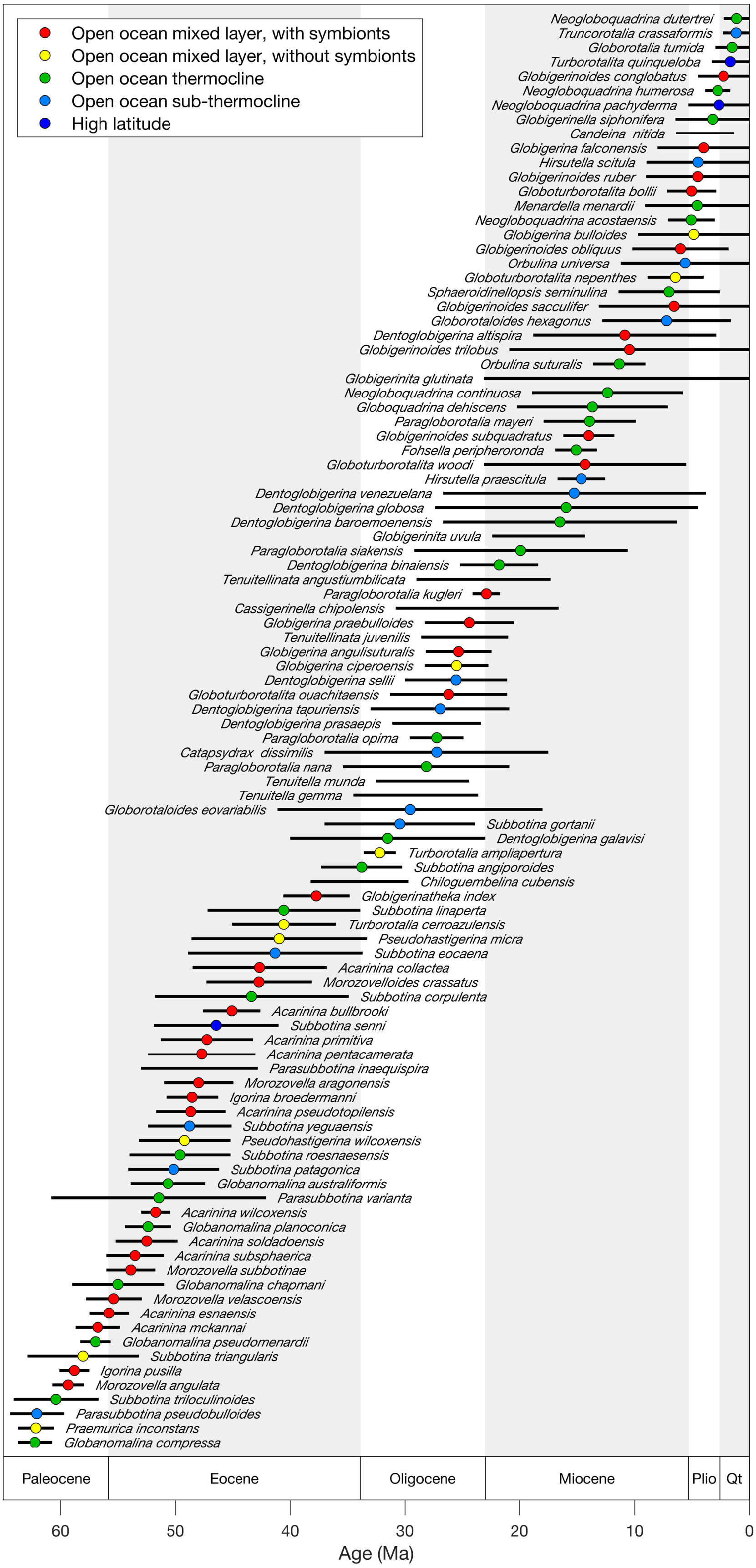

Figure S2. The 100 species of planktonic foraminifera included in the SCOR ensemble. Original NSB taxonomic assignments were mapped to the morphospecies in ref. 30 (see table S1). Horizontal lines represent periods within which each species is sufficiently widespread to contribute to SCOR, based on the species occurrence trajectories in NSB documented in ref. 17. Colored dots represent ecogroup assignments for macroperforate species from ref. 30. Microperforate species were not assigned to any ecogroup.

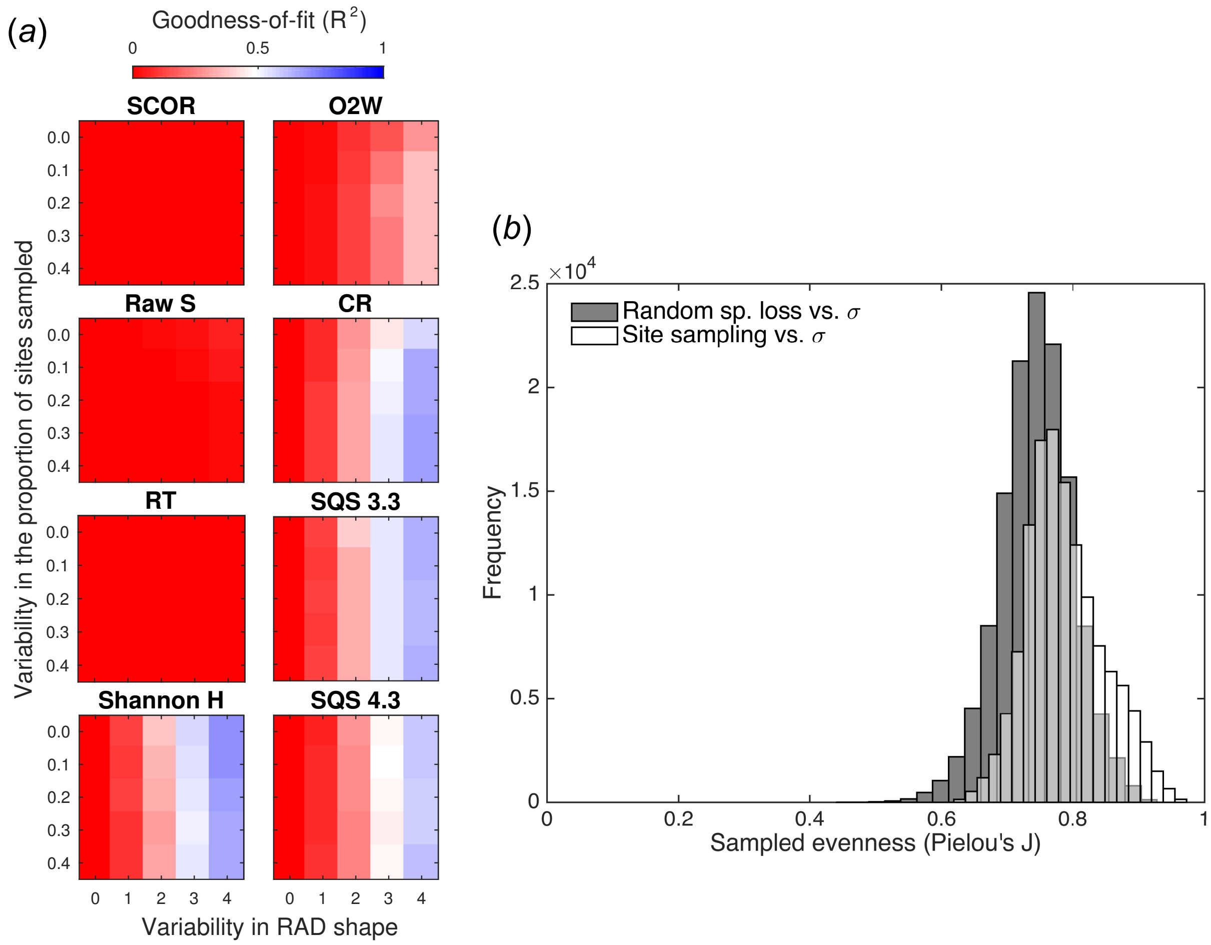

Figure S3. Subsampling methods track variability in the shape of the rank-abundance distribution. (*a*) Same experiment as in figure 1*d*, but measuring the coefficient of determination (R^2) between each estimator and the RAD shape parameter σ. Note that σ is a white noise process, and its variability is entirely random with respect to true richness and abundance. For the subsampling richness estimators, a high R^2 and blue colour here means that the estimator is effectively random with respect to the quantity it is trying to estimate. The sensitivity of subsampling methods to RAD shape thus exists regardless of how one might choose to measure sample evenness. (*b*) Distribution of sampled evenness across all Poseidon experiments. Shaded histogram represents the model runs testing the sensitivity to variability in the proportion of species randomly removed, and variability in RAD shape parameter σ (figure S5). Un-shaded histogram (note transparency in overlap) represents the model runs testing the sensitivity to variability in the proportion of sites sampled, and variability in σ (figure 1). The median evenness is 0.76, and 95% of the values are in the range 0.65 - 0.90.

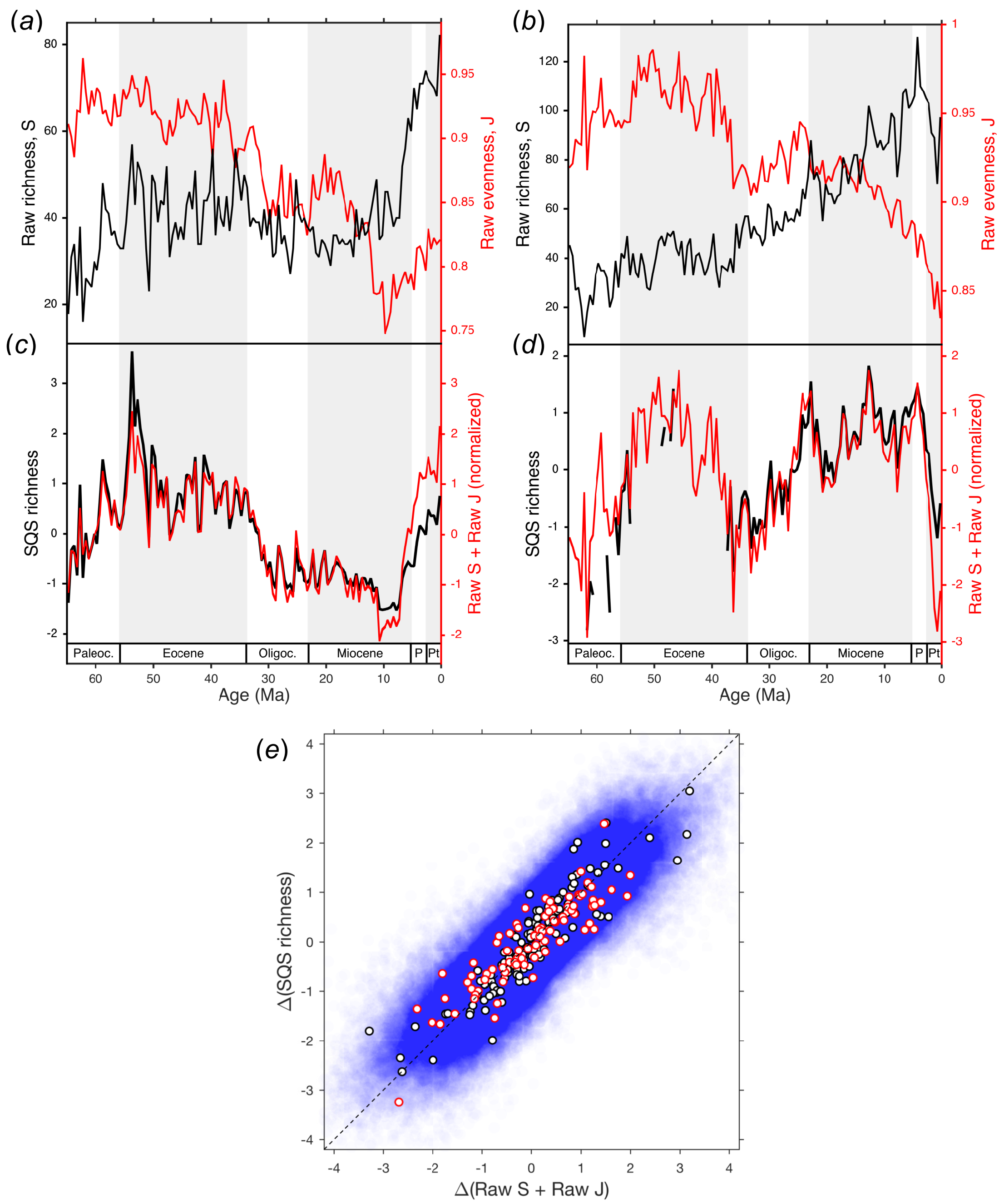

Figure S4. Sampling-standardized richness can be reproduced by the sum of raw richness and evenness. Raw sampled richness (S) and evenness (Pielou's J) of Cenozoic coccolithophores (*a*) and planktonic foraminifera (*b*) species from the NSB database. In both groups, S generally increases with the number of boreholes representing the spatial sampling, while J decreases, as expected if improved sampling enhances the detection of rare species (figure 1*b*,*c*). The sum of raw S and raw J superimposed on shareholder quorum subsampling (SQS) estimates of richness for coccolithophores (*c*) and foraminifera (*d*), all normalized to zero mean and unit standard deviation. SQS was calculated with a quorum level of 0.4 (higher quorum levels give nearly identical results but are less complete for the older part of the record). Ma, million years before present; Paleoc., Paleocene; Oligoc., Oligocene; P, Pliocene; Pt, Pleistocene. (*e*), Empirical relationship between sampling-standardized richness and the sum of raw richness and evenness. Values are first differences of normalized time series of SQS richness and of the sum of normalized raw richness (S) and evenness (Pielou's J). Data include Cenozoic coccolithophores (black) and planktonic foraminifera (red) from panels *c* and *d*, respectively, and all Poseidon model experiments (blue; N = 262,500). Stippled line marks the 1:1 relationship.

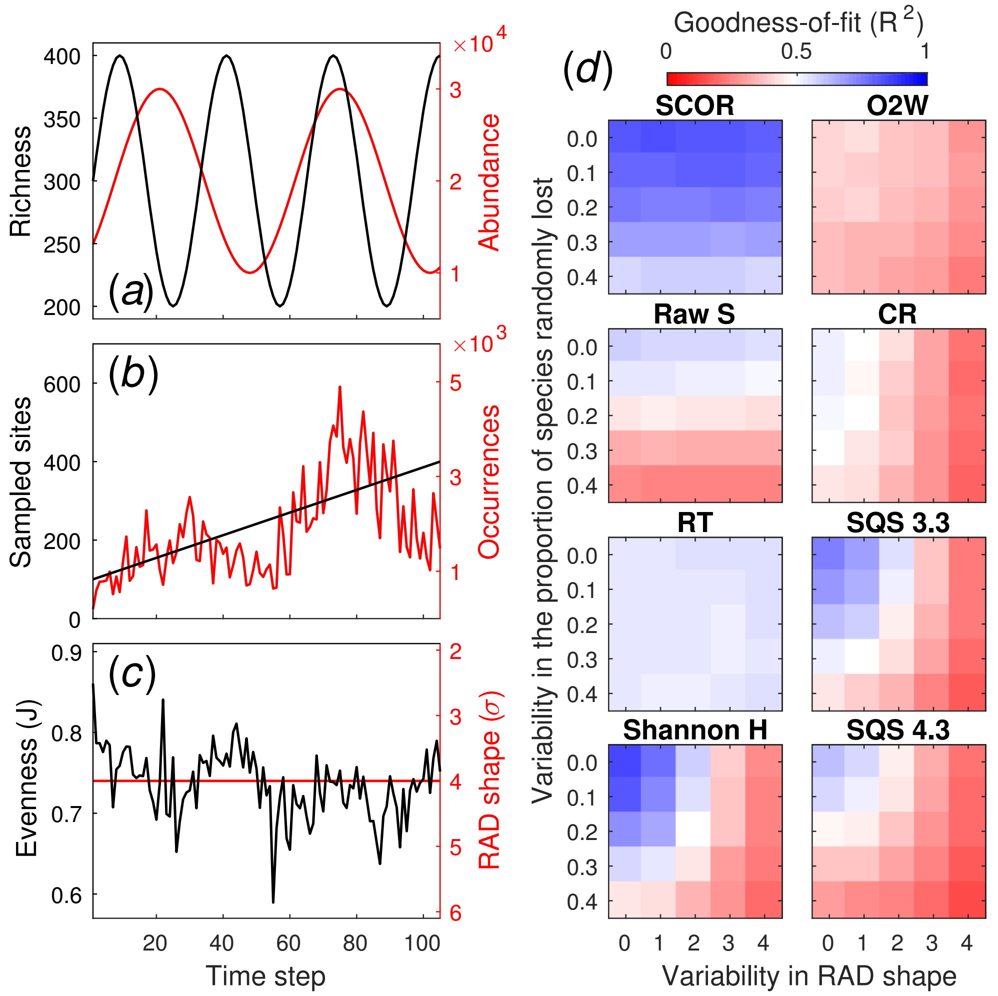

Figure S5. Effect of random species loss in Poseidon simulations. (*a*) Simulated richness and abundance as in figure 1*a*. (*b*), Site sampling (black) increases smoothly in all experiments. However, a proportion of the species is randomly removed in each time step, causing volatility in occurrences (red). This random loss could represent dissolution or other processes, such as taxonomic preferences in sample processing, that are random with respect to abundance. No variability in the proportion lost means that 50% are randomly selected and removed in each time step. In this example, variability = 0.4, meaning that between 30% and 70% of species are lost. (*c*) Even with a constant original RAD shape, the random loss of species (and variability in the proportion lost) generates volatility in sampled evenness (this example is an extreme case, see figure S3*b*). (*d*), Sensitivity to variability in RAD shape and in the proportion of species lost. Values are median goodness-of-fit (R^2) of 50 model runs, comparing SCOR to true abundance, and richness estimates to true richness.

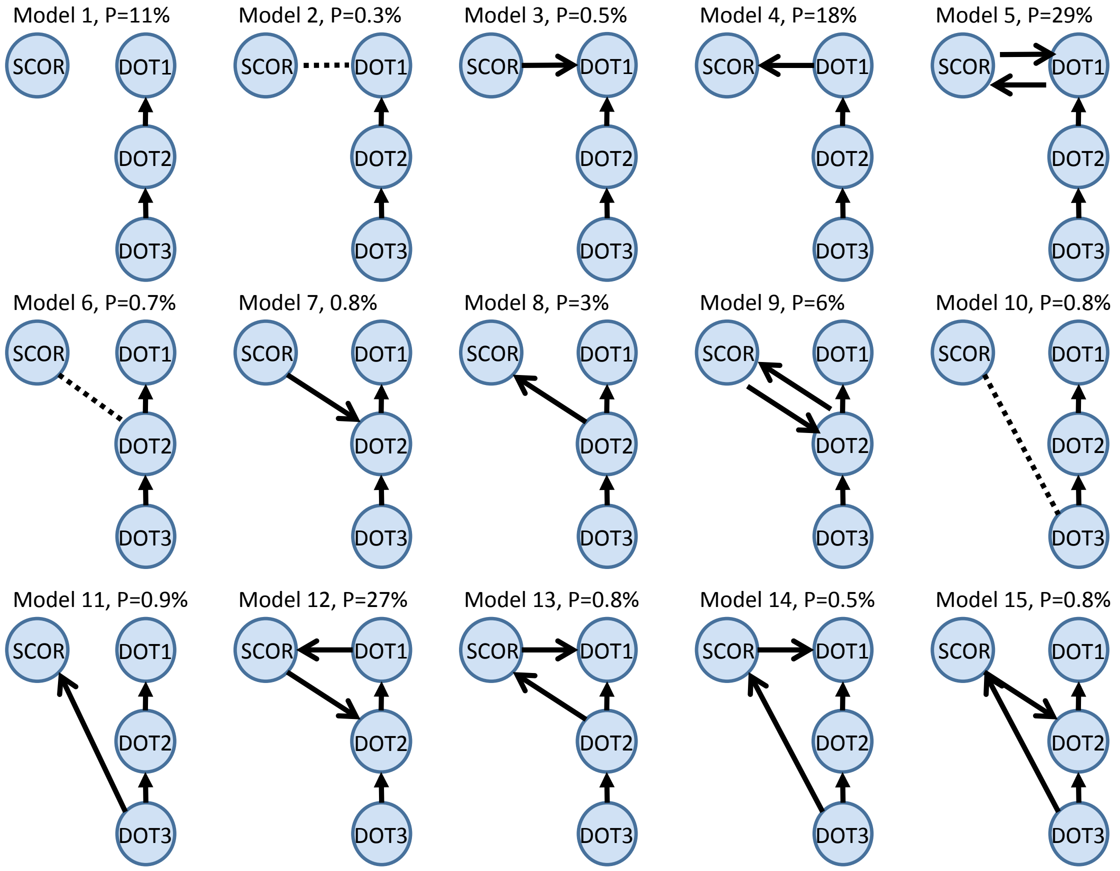

Figure S6. Schematic of all possible connection models between SCOR and DOT in linear SDE analysis. SCOR is modeled as a single-layer OUP, and DOT as a three-layer model with a WP as the lowermost layer (DOT3). Note that SCOR cannot drive DOT3 because a WP does not accept incoming causal links. Percentage values represent posterior model probabilities. Solid arrows represent casual connections pointing from driver to response (relative strength of influence not indicated here). Dotted lines represent correlative relationships. See Supplementary Text for details.

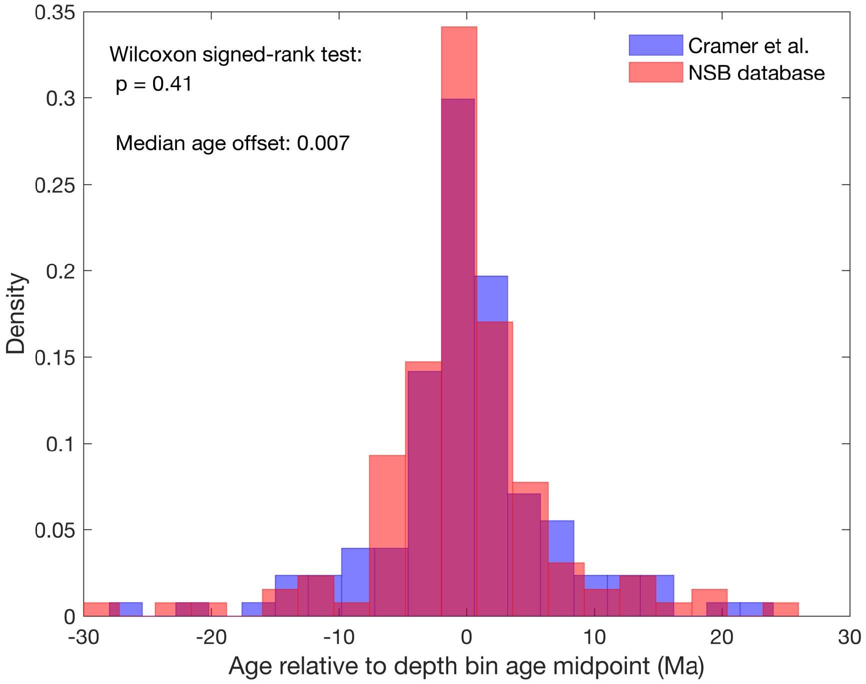

Figure S7. Testing for systematic offset between age models. A subset of 25 sites used in the NSB database planktonic foraminifera SCOR calculation were also used in the DOT reconstruction by Cramer et al. (ref. 23). To facilitate the comparison of the two age models at each site, we first discretized the overlapping sediment depth interval into depth bins, then calculated the median age for the samples in the depth bin relative to the total age range midpoint for each bin. If the age models were systematically offset, then the locations of these two binned age distributions would be shifted relative to each other. The paired differences between these two distributions give the offsets between the age models of the two databases. Although there are substantial differences between the age models, the age distributions are quite similar and symmetric, and the median offset is only 0.007 Ma. To test whether or not the two age distributions are significantly different, we performed a non-parametric Wilcoxon signed-rank test for paired differences. The null hypothesis of this test is that the difference between the pairs (i.e. the offset between the age models) follows a symmetric distribution around zero. With a test p-value of 0.41, we cannot reject the null hypothesis, suggesting that the age models are not systematically offset.

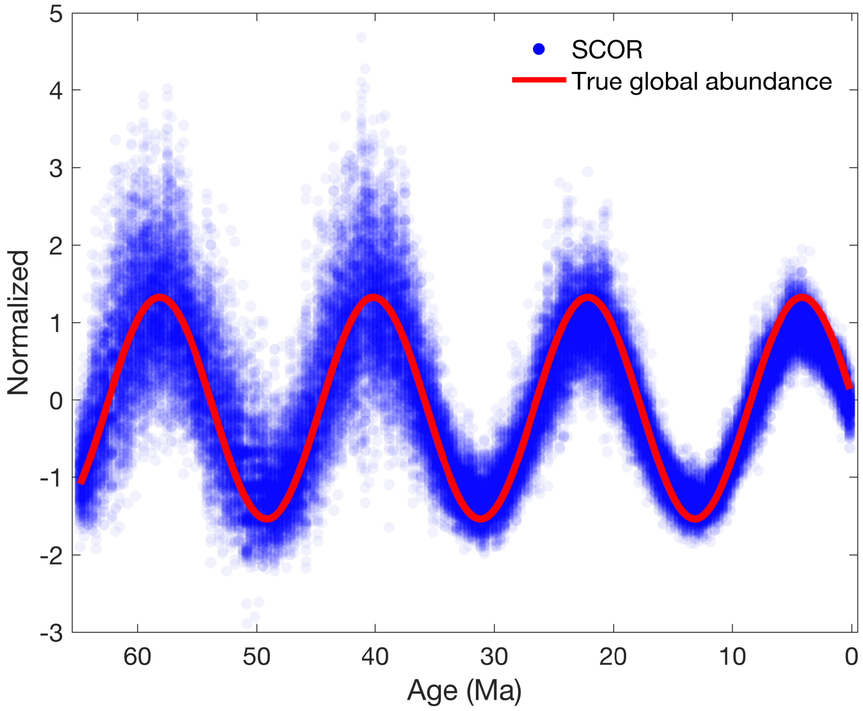

Figure S8. The effect of variable global coverage of sampled sites on SCOR. Like in all the other Poseidon simulations, species richness and total abundance vary independently (e.g. figure 1*a*). However, here we use only 500 spatial grid cells, which are assigned unique latitude-longitude coordinates to mimic the real NSB data. Hence, the total numbers of species and individuals have also been scaled down (e.g. true richness here fluctuates between 100 and 200 species), in order to limit the number of time steps in which a species is present at all sampled sites. We use 195 time steps to match the Cenozoic NSB data at 1/3 Myr resolution. Instead of randomly subsampling cells, we here sample according to the latitude-longitude coordinates of the real sampled sites in the NSB planktonic foraminifera data through the Cenozoic. Because the number and global coverage of sites decrease notably with age, SCOR shows much greater short-term volatility in the older part of the record (blue, encompassing 300 model iterations). This increased volatility is also reflected in the expanding confidence bounds on the real NSB planktonic foraminifera SCOR (figure 2*b*). Despite this reduced precision, however, SCOR is not systematically biased with respect to the long-term pattern of relative changes in true global abundance (red). Time series are normalized to mean zero and unit standard deviation.

3. Supplementary Tables

Table S1. Taxonomic re-mapping within the most common NSB species.

NSB species	Re-mapped species
Acarinina broedermanni	*Igorina broedermanni*
Acarinina intermedia	+*Acarinina esnaensis*
Acarinina rotundimarginata	+*Acarinina collactea*
Acarinina spinuloinflata	+*Acarinina bullbrooki*
Dentoglobigerina galavasi⋆	*Dentoglobigerina galavisi*
Globigerina binaiensis	*Dentoglobigerina binaiensis*
Globigerina brazieri	*Globoturborotalita brazieri*
Globigerina corpulenta	*Subbotina corpulenta*
Globigerina gortanii	*Subbotina gortanii*
Globigerina nepenthes	*Globoturborotalita nepenthes*
Globigerina ouachitaensis	*Globoturborotalita ouachitaensis*
Globigerina patagonica	*Subbotina patagonica*
Globigerina prasaepis<br>*Subbotina euapertura*	*Dentoglobigerina prasaepis*
Globigerina selli⋆	*Dentoglobigerina sellii*
Globigerina tripartita<br>*Globigerina tapuriensis*	*Dentoglobigerina tapuriensis*
Globigerina woodi	*Globoturborotalita woodi*
Globigerinatheka senni	*Subbotina senni*
Globigerinoides bollii	*Globoturborotalita bollii*
Globigerinoides quadrilobatus	+*Globigerinoides sacculifer*
Globoconella praescitula	*Hirsutella praescitula*
Globoquadrina altispira	+*Dentoglobigerina altispira*
Globoquadrina baroemoenensis	*Dentoglobigerina baroemoenensis*
Globoquadrina venezuelana	*Dentoglobigerina venezuelana*
Globorotalia crassaformis	*Truncorotalia crassaformis*
Globorotalia crassata<br>*Morozovella spinulosa*	*Morozovelloides crassatus*
Globorotalia kugleri	*Paragloborotalia kugleri*
Globorotalia menardii	+*Menardella menardii*
Globorotalia pseudomenardii	*Globanomalina pseudomenardi*
Globorotalia scitula	+*Hirsutella scitula*
Globorotaloides hexagona⋆	*Globorotaloides hexagonus*
Globorotaloides suteri	*Globorotaloides eovariabilis*
Jenkinsella mayeri	*Paragloborotalia mayeri*
Jenkinsella siakensis	*Paragloborotalia siakensis*
Paragloborotalia continuosa	*Neogloboquadrina continuosa*
Paragloborotalia pseudocontinuosa	+*Paragloborotalia nana*
Planorotalites planoconicus	*Globanomalina planoconica*
Subbotina eocaenica	*Subbotina roesnaesensis*
Subbotina inaequispira	*Parasubbotina inaequispira*
Subbotina pseudoeocaena	*Subbotina yeguaensis*
Subbotina varianta	*Parasubbotina varianta*
Tenuitella angustiumbilicata	*Tenuitellinata angustiumbilicata*
Truncorotaloides pseudotopilensis	*Acarinina pseudotopilensis*
Turborotalia compressa	*Globanomalina compressa*
Zeaglobigerina ampliapertura	*Turborotalia ampliapertura*
Zeaglobigerina labiacrassata	*Globoturborotalita labiacrassata*

Morphospecies names were re-mapped and stratigraphic distributions were checked using a combination of appendix S1 in ref. 30, the *Atlas of Eocene Planktonic Foraminifera* (ref. 32), and the available older version of the PlankRange database (ref. 31; accessed Aug. 24, 2014). A "+" indicates that both species names were present among the relatively common species in NSB and were merged. A "⋆" indicates a misspelled specific name. The following NSB species could not be matched stratigraphically with their putative synonyms and were excluded: *Acarinina rugosoaculeata*, *Globorotalia obesa*, *Globorotaloides variabilis*, *Sphaeroidinella dehiscens*, *Truncorotaloides rohri*, and *Zeaglobigerina rubescens*.

Table S2. Parameter estimates for the most probable connection model between SCOR and DOT

Parameter	Posterior median	95% credible interval
$t_{1/2,\text{SCOR}}$	0.53 Myr	(0.33, 1.1) Myr
s_{SCOR}	0.94	(0.65, 1.1)
μ_{SCOR}	0.43	(-0.01, 0.94)
$t_{1/2,\text{DOT1}}$	8.0 Myr	(0.91, 31) Myr
s_{DOT1}	1.2	(0.37, 2.4)
$t_{1/2,\text{DOT2}}$	16.0 Myr	(3.4, 166) Myr
s_{DOT2}	0.45	(0.03, 2.8)
σ_{DOT3}	0.027	(0.003, 0.22)
$\beta_{\text{DOT1}\rightarrow\text{SCOR}}$	0.45	(0.14, 0.76)
$\beta_{\text{SCOR}\rightarrow\text{DOT1}}$	-0.77	(-2.3, 0.76)
$t_{1/2,\text{uSCOR1}}$	0.91 Myr	(0.32, 1.6) Myr
s_{uSCOR1}	0.07	(0.01, 0.35)
$t_{1/2,\text{uSCOR2}}$	2.4 Myr	(0.86, 68) Myr
s_{uSCOR2}	0.94	(0.65, 1.1)
σ_{uSCOR3}	2.8	(0.04, 5.8)
$t_{1/2,\text{uDOT1}}$	0.24 Myr	(0.13, 0.33) Myr
$t_{1/2,\text{uDOT2}}$	0.50 Myr	(0.21, 1.5) Myr
σ_{uDOT3}	4.1	(1.9, 8.9)
$\beta_{\text{uDOT1}\rightarrow\text{uSCOR1}}$	2.7	(-2.6, 3.8)
$\beta_{\text{uSCOR1}\rightarrow\text{uDOT1}}$	-2.7	(-5.2, 1.9)

Parameter estimates for the most probable model in SDE analysis, where $t_{1/2,i}$ are half-lives, s_i are stationary standard deviations, μ_i are the expected values of OUPs, σ_i are stochastic terms in WPs, and β_i are connection strengths. For the pre-processed data, the lowermost DOT layer (DOT3) is a WP, and the most probable model corresponds to Model 5 (figure S6). The interpretation of parameter values depends on other processes, e.g. the causal connection from DOT to SCOR will increase the total stationary standard deviation of the SCOR process. The reported values represent what each process (layer) itself supplies in isolation. For untransformed data (uDOT and uSCOR), both uDOT and uSCOR have three-layer models with a lowermost WP. See Supplementary Text for details.

Additional supplementary material

Datasets

The SCOR and DOT data are available in a separate Excel file.

Code

R code for Poseidon simulations are available on GitHub: https://github.com/bhannis/Poseidon
